# Pollinator-Friendly Policies in Brazil: History and Future Directions

**DOI:** 10.1007/s13744-025-01355-6

**Published:** 2026-04-23

**Authors:** Juliana Hipólito, Carmen Sílvia Soares Pires, Jeferson Gabriel da Encarnação Coutinho, Braulio Ferreira de Souza Dias

**Affiliations:** 1 National Institute of Science and Technology in Pollination: Knowledge, Conservation, and Sustainable Use of Pollinators (INCT INPol), Rio de Janeiro, Brazil; 2 Instituto Nacional da Mata Atlântica (INMA), Santa Teresa, ES Brazil; 3 Ecology and Biosafety Laboratory, Embrapa Recursos Genéticos E Biotecnologia, Brasília, DF Brazil; 4 Empresa Brasileira de Pesquisa Agropecuária, Embrapa Maranhão, São Luís, Maranhão, Brazil; 5 Ecology Department, University of Brasilia, Brasília, Brazil; 6 Ministry of the Environment and Climate Change, Brasilia, Brazil

**Keywords:** Bees, Pollinators conservation, Nature conservation, Agrochemicals impacts

## Abstract

This article reviews existing and proposed Brazilian policies aimed at promoting the conservation and sustainable use of wild and managed pollinators. Emphasis is placed on strategies for habitat conservation and restoration, as well as the reduction of stress associated with agrochemical use. The origins and development of the International Pollinators Initiative (IPI) under the United Nations Convention on Biological Diversity (CBD) and the Brazilian Pollinators Initiative are detailed, including their connections to pollinator and pollination assessments conducted by the Intergovernmental Science-Policy Platform on Biodiversity and Ecosystem Services (IPBES) and its national counterpart, the Brazilian Platform on Biodiversity and Ecosystem Services (BPBES). A summary of Brazil’s current nature conservation policies, particularly the Native Vegetation Protection Law (the updated version of the Brazilian Forest Code), is provided, alongside policies that promote sustainable agriculture. The article also presents a concise review of the usage and impacts of agrochemicals in Brazil on both humans and pollinators, with a focus on bees, and discusses the prospects for biological control. The paper concludes by outlining critical areas requiring greater attention from public policies.

## Introduction: The pollination crisis

The economic benefit of animal to crops has been estimated at approximately from US$195 billion to US$387 billion (Gallai et al. 2009; Calderone 2012; Wolowski et al. 2019; Porto et al. 2020; Oliveira et al. 2024). Furthermore, over 95% of wild plants globally depend on animal pollination for their reproduction, population survival, and adaptation (IPBES 2016a, b; Imperatriz-Fonseca et al. 2012, 2019; Rech et al. 2014; Gemmil-Herren 2016; Wolowski et al. 2019). Despite this critical role, the world faces an accelerating decline in pollinator populations and an increasing pollination deficit, affecting both wild plants in fragmented ecosystems and cultivated crops (IPBES 2016a, b; Wolowski et al. 2019). This decline poses a severe threat not only to biodiversity but also to the global economy and food security.

The decline in pollinator populations is driven by multiple, interacting factors. On the one hand, there is extensive degradation of natural ecosystems due to ongoing expansion of areas dedicated to crops, livestock, aquaculture, single‑species forest plantations, mining, hydropower plants, urbanization, and infrastructure development (FAO 2020a). On the other hand, the intensification of production systems involves increased agrochemical use and heightened exposure of managed bees to stress and diseases (Biesmeijer 2006; Butchart et al. 2010; Potts et al. 2010; Goulson et al. 2015; Aizen et al. 2019).

Beyond honey bees and bumble bees, solitary wild bees are also threatened, and their populations are declining in many regions (e.g., Biesmeijer et al. 2006; Nieto et al. 2014; Goulson et al. 2015). The continuous collapse of bumble bee populations in recent decades (Nieto et al. 2014) and the high mortality rates of honey bee colonies, including instances of Colony Collapse Disorder (CCD), over the past two decades (Ellis et al. 2010; Pettis et al. 2013; DeGrandi‑Hoffman & Chen 2015; Laurent et al. 2015, Gray et al. 2023), have significantly elevated attention to this issue.

Yet, most focus on pollinators remains predominantly centered on bee species. Ecological crop and wild plant reproduction relies heavily on diverse animal taxa, including various insect orders like Lepidoptera (e.g., butterflies, moths), Coleoptera (beetles), Diptera (flies), and other Hymenoptera (e.g., wasps), alongside vertebrates such as bats, birds, and even some non-volant mammals (Fleming et al. 2009; Rader et al. 2016; Ratto et al. 2018). These groups offer unique functional services crucial for agricultural output and ecosystem health, often irreplaceable by bees alone. Evidence, such as Lewinsohn et al.’s (2022) Brazilian appraisal noting widespread terrestrial insect reductions, including butterflies and scarab beetles, confirms a broader pattern of insect loss affecting varied species vital for these services.

This disproportionate research focus risks a substantial underestimation of true global pollination deficits, especially where non-bee taxa are dominant or critical. Neglecting the status of these diverse species leaves ecological functions and agricultural systems vulnerable and poorly understood, thereby weakening conservation strategies. While continued research into bee health is crucial, broadening monitoring and scientific inquiry to encompass the full spectrum of animal groups providing these services is essential for robust food security and effective ecosystem management. Given the predominant focus in existing literature, our investigation primarily utilized studies centered on bees. However, we underscore the critical importance of future research encompassing a broader spectrum of animal pollinators to capture the full ecological complexity.

Recent studies have provided robust evidence of the environmental impacts of neonicotinoid pesticides (Goulson 2013; Wood & Goulson 2017), demonstrating that acute (typically single/short exposures with effects assessed within ≈ 24–96 h) or chronic exposure (repeated exposures over days/weeks) to these pesticides alone can significantly alter honey bee flight and impair foraging and homing behaviors, which are vital for normal colony function and ecosystem services (Tosi et al. 2017a, b). Furthermore, studies have shown that stingless bees are also negatively affected by sub‑lethal pesticide exposure (Lima et al. 2016; Dorneles et al. 2017; Miotelo et al. 2021). While *Apis mellifera* is typically used as the model for pesticide risk assessment (Botina et al. 2024; Bernardes et al. 2022), there is a clear need for more extensive studies on native bees in this context (Cham et al. 2019; Miotelo et al. 2021; Lourencetti et al. 2023a, b).

### Policy and governance frameworks

In (Ipbes 1996), Buchmann and Nabhan published the influential book “The Forgotten Pollinators” (Buchmann & Nabhan 1996), which inspired the successful North American Pollinator Campaign and prompted the Ministério do Meio Ambiente e Mudança do Clima—MMA (n.d.) (*Ministry of Environment and Climate Change*) and the Empresa Brasileira de Pesquisa Agropecuária—Embrapa (*Brazilian Agricultural Research Corporation*) to prioritize pollinator conservation. This topic was integrated into a proposed work program on sustainable agriculture under the *Biodiversity Diversity Convention Convenção sobre Diversidade Biológica*—CBD), adopted at COP 3 in 1996 as Decision III/11 on the conservation and sustainable use of agricultural biological diversity (Campanhola et al. 1998; Dias 2021a, b).

In 1998, an international workshop coordinated by B. F. S. Dias, with support from V. L. Imperatriz‑Fonseca and A. Raw, gathered 60 experts at the University of São Paulo and produced the São Paulo Declaration on Pollinators, recommending the establishment of an international initiative for the conservation and sustainable use of pollinators in agriculture, with emphasis on bees (Dias et al. 1999; Kevan & Imperatriz‑Fonseca 2002; Imperatriz-Fonseca et al. 2004). This initiative was subsequently adopted under the CDB in Decision V/5 in 2000, with the Food and Agriculture Organization of the United Nations—FAO invited to coordinate implementation. In 2012, the *Intergovernmental Platform on Biodiversity and Ecosystem Services *—IPBES was established by the United Nations Environment Programme—UNEP (Programa das Nações Unidas para o Meio Ambiente; PNUMA). Operating similarly to the IPCC, IPBES provides critical assessments to inform decision‑making at international, national, and local levels. In 2016 and 2017, IPBES published its Assessment Report on Pollinators, Pollination and Food Production (IPBES 2016a, b), which informed CDB policy decision XIII/15 (2016) and, subsequently, XIV/6 (2018) (CBD Secretariat 2016a, b, 2018). Following IPBES recommendations, the Coalition of the Willing on Pollinators (Promote Pollinators n.d.) was founded in 2016 at CBD COP 13 to support national strategies for pollinator conservation. Over 30 countries have joined; Brazil, however, has not yet formalized membership.

Also in 2016, during the II Brazilian Symposium on Pollination, the Brazilian Network of Plant–Pollinator Interactions – REBIPP (Rede Brasileira de Interações Planta-Polinizador) was established. This effort was bolstered by the GEF‑supported Projeto Polinizadores do Brasil (2010–2014), which laid the groundwork for current actions and contributed to the REBIPP database. Two years later, at CBD COP 14 (2018), Parties adopted the Plan of Action 2018–2030 for the International Initiative for the Conservation and Sustainable Use of Pollinators, aiming to coordinate efforts to safeguard wild and managed pollinators and foster the sustainable use of pollination services (CBD Secretariat 2018; Dias 2021a, b). The Plan also emphasized the engagement of Indigenous People, local communities, and other relevant actors. More recently, CBD COP 15 (CBD 2022) adopted Decision 15/4 establishing the Kunming–Montreal Global Biodiversity Framework, with four long‑term goals (2050) and 23 action targets (2030), including ecosystem conservation and restoration, halting species extinctions, ensuring sustainable management of native and domesticated species, promoting sustainable agriculture, and maintaining ecosystem services with explicit reference to pollination.

Within Brazil, in 2019, REBIPP and the Brazilian Platform on Biodiversity and Ecosystem Services—BPBES (produced the first comprehensive national report on pollination, pollinators, and food production (Wolowski et al. 2019), which drew attention to the Brazilian context and motivated the consultation process for the Plano de Ação Nacional para a Conservação de Insetos Polinizadores—PAN Insetos Polinizadores (National Action Plan for the Conservation of Pollinating Insects), coordinated by the Instituto Chico Mendes de Conservação da Biodiversidade—ICMBio (Chico Mendes Institute for Biodiversity Conservation) and launched in late 2022. Despite being initiated in 2019, this Action Plan remains in preparation.

In this context, several important publications have emerged over the past decade, including manuals providing guidance on pollinator management in major Brazilian crops. These materials were developed under the "Project Conservation and Management of Pollinators for Sustainable Agriculture through an Ecosystem Approach" (GEF‑FAO‑UNEP; in Brazil, coordinated by the MMA with managerial support from the Fundo Brasileiro para a Biodiversidade—FUNBIO (Brazilian Biodiversity Fund). Additional contributions came from the PROBIO (Projeto de Conservação e Uso Sustentável da Biodiversidade Brasileira—*Project for the Conservation and Sustainable Use of Brazilian Biodiversity*) I; a call in 2009 from CNPq (Conselho Nacional de Desenvolvimento Científico e Tecnológico—*National Council for Scientific and Technological Development*); and an initiative coordinated by the University of São Paulo). CNPq and the Associação A.B.E.L.H.A (Associação Brasileira de Estudo das Abelhas—Brazilian Association for the Study of Bees) issued research calls in 2018 and 2021 (last call). Manuals and research published between 2003 and the present indicate a consolidated knowledge base that integrates traditional and scientific knowledge; best agricultural practices for pollinator conservation and sustainable use; enhanced capacity; and increased awareness among the public and policymakers (MMA online publications; Yamamoto et al. 2014; Witter et al. 2014; Dias 2006; Imperatriz‑Fonseca et al. 2012; Rech et al. 2014; Giannini et al. 2015; Klein et al. 2020). A major step toward consolidating Brazil’s pollination research network was the approval and funding in 2023 of the Instituto Nacional de Ciência e Tecnologia (National Institute of Science and Technology) “INCT—Pollination: knowledge, conservation and sustainable use of pollinators” by CNPq.

Despite recent advancements, there is broad consensus that current public policies remain inadequate to effectively address pollinator‑related issues (Byrne & Fitzpatrick 2009; Dicks et al. 2016; Hall & Steiner 2019; Hipólito et al. 2021). This inadequacy spans: (i) the diversity of pollinators and plant dependency on animal pollination; (ii) pollination of crops and wild plants and their economic values; (iii) protection and restoration of habitat diversity (forage, nesting substrates); (iv) pesticide control (acute and chronic effects, adherence to IPM); v) integrated pest and pollinator management (IPPM) (Biddinger & Rajotte 2015); (vi) treatment of pollinators as bio‑inputs in agriculture; (vii) control of invasive alien species and associated pests and diseases; (viii) pollinator health and sanitary measures for bees and other pollinators; (ix) pollinator population collapse and extinction (monitoring, red listing, and action planning); (x) sustainable use of managed pollinator species (honey bee, bumble bees, stingless bees, solitary bees); (xi) certification and economic incentives; (xii) citizen science monitoring; (xiii) research and technology development; (xiv) inclusion of pollination in school curricula; and (xv) public education and farmer extension services.

Broadly, there is a pressing need for public policies that promote pollinator and habitat protection and restoration, reduce or eliminate threats, and enhance the availability of pollination services for both cultivated and wild plants. Prior works have reviewed or proposed policy options abroad (Eardley et al. 2006; Tang et al. 2007; Byrne & Fitzpatrick 2009; Mader et al. 2011; Rose et al. 2015; Dicks et al. 2016; Gemmil‑Herren 2016; Schelske et al. 2018) and in Brazil (Cunha & Landeiro 2012; Freitas & Bomfim 2017; Pires et al. 2016; Dos Santos et al. 2018; Hipólito et al. 2021).

In July 2013, the Chamber of Deputies (Câmara Dos Deputados 2013) and, in August 2013 and March 2018, the Federal Senate (Senado Federal 2013, 2018) organized public hearings on honey bee mortality and risks to pollination services. Bill 1634 (2007), by Deputy João Dado (PDT‑SP), aimed to introduce measures for the protection of bees and associated plants. A Senate hearing led to Bill 1918/2019 (Senator Lasier Martins, PODE/RS), proposing legal measures to protect pollinators. As of May 2022, these bills remained pending and were ultimately archived due to lack of voting. Of three Senate bills related to bees, only Bill 1918/2019 specifically addressed measures to stimulate research and pollinator protection; others focused on policies encouraging honey production and apicultural products and services (Bill 6913/2017) or inspection and regulation of agricultural products like honey (Bill 3358/2015). Hipólito et al. (2021) reviewed Brazilian pollinator‑relevant policies (federal and state) and concluded that specific laws for pollinator maintenance are absent, and that existing policies generally lack necessary standards for sustainable conservation underscoring the need for more comprehensive, interdisciplinary legislation. This article builds on that context, emphasizing key areas requiring attention and considering international examples relevant to Brazil.

### Habitats conservation and restoration

**Fig. 1:**
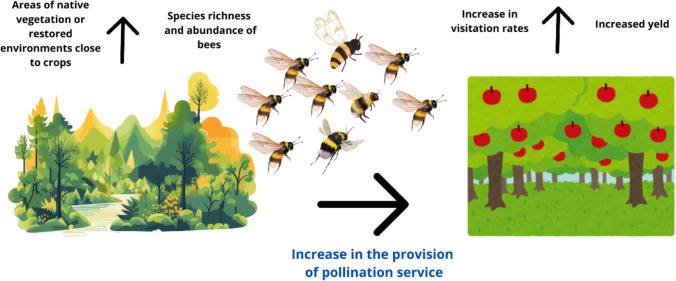
The presence of native vegetation and restored areas near crops increases the availability of nesting sites and trophic resources for bees, increasing species richness and abundance (FAO 2020b). When these areas are close to agricultural crops, the chance of providing pollination services increases, resulting in higher crop productivity. Source: Authors

The establishment and management of protected areas and other effective area-based conservation measures (OECMs, IUCN 2019) play a pivotal role in maintaining pollinator-rich natural or semi-natural ecosystems. These areas provide unique research opportunities to understand natural pollination syndromes and promote abundant pollination services to wild plant communities and to small-scale agriculture practiced by Indigenous Peoples and local communities. Additionally, protected areas and OECMs can deliver adequate levels of pollination services to neighboring croplands within the flight range of pollinators (Fig. 1). Flight ranges vary substantially, from a few hundred meters for most small bees (including stingless bees), flies, beetles, and butterflies, to several kilometers for larger bees and moths, hummingbirds, and bats (Carvalheiro et al. 2010; Zurbuchen et al. 2010a, b; Benjamin et al. 2014). However, most crop areas in Brazil are located far from existing protected areas and OECMs and, therefore, do not directly benefit from them for their pollination needs (Giannini et al. 2020).

One of the key objectives of the IUCN Bonn Challenge is to restore 350 million hectares of degraded land by 2030 (Temperton et al. 2019). The maintenance of restored plant communities requires sustaining diverse pollinator communities, especially bees, within these landscapes (Menz et al. 2011; FAO 2020b). Landscapes featuring diverse habitats characterized by a range of plant species, land cover types, and seasonal resource availability are more likely to support bees with a wide array of functional traits (Coutinho et al. 2021). This is because social and solitary bees, ground- and cavity-nesting bees, and bees with different tongue lengths and body sizes utilize environmental resources in distinct ways. Moreover, while multiple studies report positive associations between functional diversity and ecosystem services (e.g., Wood et al. 2015; Hipólito et al. 2018; Dainese et al. 2019; Woodcock et al. 2019), evidence indicates that benefits to crop production can be context dependent and not universally guaranteed. Thus, for successful restoration, managing for functionally diverse pollinator assemblages is advisable, while recognizing variability across cultivated species, crop systems, and landscapes.

Several actions can promote higher bee diversity at farm and landscape scales. For example, uncommon native bee species were sevenfold more abundant on hedgerow flowers than at weedy, unmanaged edges; 40% of species observed on hedgerow flowers were exclusive to hedgerow sites. Hedgerows are especially important for supporting less common native bees in intensive agricultural landscapes (Morandin & Kremen 2013). Similarly, wildflower plantings adjacent to crop fields can increase the abundance of wild pollinators during crop bloom, enhancing pollination services and yield (Blaauw & Isaacs 2014). Providing forage habitat near pollinator dependent crops is, therefore, an effective strategy to conserve wild pollinators in otherwise resource-poor agricultural environments.

Brazilian biomes face multiple environmental challenges that threaten pollinator communities (Joly et al. 2019). Deforestation, intensive farming, widespread agrochemical use, mining, desertification, large-scale infrastructure projects, uncontrolled burning, and the scarcity of designated conservation areas are key drivers of impacts on pollinators. Deforestation causes both habitat loss and fragmentation. Habitat loss reduces vegetation cover and the quantity/quality of floral resources for bees and other pollinators (Kennedy et al. 2013), removes hollow trees, and increases soil compaction, reducing nesting sites. Fragmentation can impede movement across the landscape, lowering pollen flow among remnants and, in plant species dependent on animal pollination, decreasing genetic diversity (Aguilar et al. 2006).

For pollinators, species with limited mobility may become confined to isolated habitat patches, compromising their long-term viability. Beyond the typical problems associated with reduced genetic variability in small, isolated populations, bees also face a specific issue: increased homozygosity at sex determination loci can lead to the production of diploid males; workers may eliminate queens in such cases, weakening colonies and potentially leading to colony failure (ICMBIO 2018). Conversely, heterogeneous, connected, and pollinator-friendly landscapes support the maintenance and growth of pollinator populations by providing trophic resources and nesting sites and contribute to more stable pollination services (Viana et al. 2012; Ferreira et al. 2015).

Accordingly, there is a clear need for public policies, including economic incentives, that promote the conservation or restoration of natural and semi-natural ecosystems within agricultural landscapes and on farms. In Brazil, the Lei de Proteção da Vegetação Nativa — LPVN (Native Vegetation Protection Law; updated Brazilian Forest Code; Law 12.651/2012) establishes requirements for the conservation of natural vegetation on private lands, including Áreas de Preservação Permanente—APPs (Permanent Preservation Areas) and Reservas Legais—RLs (Legal Reserves). Internationally, examples include conservation requirements linked to public land concessions (e.g., USA and Australia) and economic incentives embedded in agricultural subsidies such as the US Farm Bill and the EU Common Agricultural Policy—CAP (Política Agrícola Comum da União Europeia). More recently in Brazil, sustainable agriculture policies and programs—e.g., Sistemas de Produção Integrada de Alimentos (Integrated Food Production Systems) (MAPA 2009), Sistema Plantio Direto—SPD (No Tillage Cropping System) (Landers 2005), Integração Lavoura Pecuária Floresta—ILPF (Crop Livestock Forest Integration), o Plano de Agricultura de Baixa Emissão de Carbono—Plano ABC/ABC + (Low Carbon Emission Agriculture Plan) (Lima et al. 2020; MAPA 2021), na the Política Nacional de Agroecologia e Produção Orgânica—PNAPO (n.d.) (National Policy on Agroecology and Organic Production), as well as recent proposals to provide lower interest rates within the Plano Safra (Brazilian Farm Bill) for farmers adopting sustainable practices, play a vital role. The relevance of these policies to pollinators lies in their potential to increase the year-round availability of foraging resources (nectar, pollen, floral oils, resins) and nesting substrates (tree and branch cavities, suitable soils, ravines, and similar habitats).

Another broad conservation approach supported by public policies involves species-focused measures: regular assessments of conservation status and monitoring of pollinators; formal inclusion of threatened species in red lists and red data books/databases; legal protection; and conservation action plans (e.g., Planos de Ação Nacional — PANs e Planos de Ação Territoriais—PATs) to restore threatened populations. Conservation assessments are coordinated globally by IUCN (via the Species Survival Commission), regionally (e.g., European Union), nationally and sub-nationally (in Brazil, by the ICMBio). Among pollinator groups, regular assessments are currently available mainly for bees, butterflies, birds, and bats. For bees in Brazil, of 209 species assessed between 2009 and 2014, 4 were classified as Endangered, 1 as Vulnerable, 2 as Near Threatened, 180 as Least Concern, and 22 as Data Deficient (ICMBIO 2018, volume 7). Habitat loss and alteration from agricultural activities and urban expansion are the primary pressures. Substantial gaps remain for many pollinator groups, underscoring the need to invest in baseline surveys across all Brazilian biomes, including non-bee pollinators that are often neglected (Rader et al. 2016; Lopes et al. 2021).

### Pesticide use in Brazil

The average annual impact of pests on Brazilian agriculture is estimated at 7.7% of crop yield (≈ US$ 14.7 billion per year; Oliveira et al. 2014), which is lower than the global average loss of 13–13.8% (Pimentel 1986). Expenditure on pesticides in Brazil in 2011 was US$ 8.5 billion (164,074 metric tons of pesticides; Oliveira et al. 2014). In 2013, 102 active ingredients and 423 commercial products were available on the market; 52% were toxicity classes I (highly hazardous) and II (very hazardous). Brazil is currently the second largest overall consumer of agrochemicals in absolute values, showing a strong increase between 2009 and 2013 and stabilizing thereafter, with an apparent average annual consumption of around 755,489 metric tons of active ingredients and average use of 4.3 kg/ha (according to the Ministério da Agricultura e Pecuária—MAPA, Ministry of Agriculture and Livestock; MAPA), 6.2 kg/ha (Zhang 2018), or 8.3 kg/ha, reaching 12–16 kg/ha in the states of Mato Grosso, Mato Grosso do Sul, Goiás, and São Paulo (Bombardi 2017). The herbicide glyphosate represents a substantial portion of this consumption: according to the Instituto Brasileiro do Meio Ambiente e dos Recursos Naturais Renováveis—IBAMA (Brazilian Institute of Environment and Renewable Natural Resources; IBAMA), from 2009 to 2020, sales of glyphosate (tons of active ingredient) accounted for approximately 60% of all pesticide sales, making it the most sold active ingredient in Brazil during this period among the 309 active ingredients listed in the 2020 Bulletin of Production, Import, Export, and Sales of Agrochemicals in Brazil (IBAMA 2020).

Herbicide consumption in Brazil increased dramatically following the approval of glyphosate-resistant soybean in 2005, rising from 22,903 metric tons in 1990 to 413,833 metric tons in 2020 (FAO 2019; FAO 2022). Glyphosate use alone surged 75% from 2009 to 2019, while total pesticide use expanded from 141,130 tonnes in 2000 to 685,746 tonnes by 2020 (Merotto et al. 2022; Procópio et al. 2024). By 2022, Brazil became the world’s largest herbicide consumer with 492,450 metric tons annually, surpassing the United States (405,500 metric tons) (FAO 2022). This escalation far exceeds growth in cultivated area, reflecting intensified chemical dependence driven by no-tillage adoption and herbicide-resistant crop technologies (Procópio et al. 2024). Concurrent with market expansion, herbicide costs per hectare increased, driven by both increased application rates and newer, more expensive active ingredients required to manage resistant weed populations (Merotto et al. 2022). Glyphosate application frequency intensified from 1.8 to 2.4 applications per season between 2005/06 and 2010/11, exemplifying this trend (Merotto et al. 2022). Approximately 90% of herbicide sales are financed through agrochemical company credit (~ 250 days), compared to only 10% from public funds, fostering farmer loyalty and accelerating technology adoption (Santos, 2012). These trends underscore a critical paradox: while herbicide intensification has sustained production growth, it has simultaneously created a cycle of escalating chemical dependency and herbicide resistance.

Production and consumption of agrochemicals in Brazil have received economic incentives since the 1960s. "Sistema Nacional de Crédito Rural"—SNCR (National Rural Credit System; SNCR), created in 1965, is highlighted as a fundamental factor for the growth in agrochemical consumption, by conditioning access to public agricultural credit on compulsory purchase of chemical inputs by farmers (Silva et al. 2005). Another initiative was the "Programa Nacional de Defensivos Agrícolas" (National Program of Agricultural Defenses), created in 1975 under the II "Plano Nacional de Desenvolvimento"—II PND (Second National Development Plan), which provided financial resources for the creation of domestic enterprises and for the establishment in Brazil of subsidiaries of transnational agricultural input companies (De Benedicto et al. 2019). Additionally, agrochemicals receive tax benefits: exemption from the Imposto sobre Produtos Industrializados—IPI (Industrialized Products Tax) (Decree 6.006, Dec 26, 2006); 60% reduction in the Imposto sobre Circulação de Mercadorias e Serviços—ICMS (State Value Added Tax) (ICMS Agreement 100, Nov 6, 1997); and exemption from PIS/PASEP contributions (Decree 5.630, Dec 22, 2005), with complementary exemptions in some states (Londres 2011; De Benedicto et al. 2019; Mitidiero Junior & Goldfarb 2021).

Additionally, all agrochemicals in Brazil are exempt from the Imposto sobre Produtos Industrializados—IPI (Industrialized Products Tax; IPI) (Federal Decree No. 6.006, December 26, 2006), receive a 60% reduction in the ICMS (ICMS Agreement 100, November 6, 1997), and are exempt from PIS/PASEPcontributions (Federal Decree No. 5.630, December 22, 2005). In addition to federal tax relief, some Brazilian states grant complementary exemptions (Londres 2011; De Benedicto et al. 2019; Mitidiero Junior & Goldfarb 2021).

A review on the safe use of agrochemicals in Brazil (2000–2014; Abreu & Alonzo 2014), based on 25 detailed studies, found that 65–89% of interviewed users did not follow agronomic prescriptions as required by the Lei de Agrotóxicos—Lei No. 7.802/1989 (Brazilian Pesticides Law), instead relying on advice from neighbors or vendors rather than independent agronomists. Combined with evidence that ≈ 90% of sales are made directly by agrochemical companies, this suggests that many farmers follow company technical guidance for purchase and use. This pattern helps explain the rapid increase in seed coatings with neonicotinoids and herbicides associated with herbicide-tolerant genetically modified crops, which conflicts with a central Integrated Pest Management—IPM principle: monitoring to decide whether to apply a control product. IPM was more prevalent in Brazil before the sharp rise in agrochemical consumption observed in the last two decades. Moreover, in vast monocultures (e.g., continuous areas of 5000–10,000 ha of soybean, corn, or cotton), it is practically difficult to implement timely monitoring as a decision tool; preventive, calendar-based spraying is common. Another major issue is the illegal market: smuggled products may represent about 20% of total sales, including non-authorized products lacking proper risk assessment (de Moraes 2022). A comparison of nationwide pest lists since the 1930 s indicates a steady increase in the number of pests per crop species, interpreted by Paschoal (1979) as consistent with selection for pesticide resistance.

Despite the lack of comprehensive, standardized monitoring of pollinator populations in Latin America, surveys have reported high honey bee colony losses (Requier et al. 2018, 2024). In Brazil, high rates of honey bee mortality and colony loss have been reported mainly in the Southeast and South (Pires et al. 2016; Castilhos 2018; Freitas et al. 2017; Castilhos et al. 2019; Dias De Freitas et al. 2022), and, where investigated, these cases were associated with pesticide contamination rather than pathogens or parasites. In the first half of 2019, press and digital media reported losses exceeding half a billion honey bees in Southern Brazil. Unsurprisingly, Dos Santos et al. (2018) documented beekeepers’ reluctance to provide hives of honey bees and stingless bees for crop pollination due to heavy pesticide use and concerns over bee health.

**Fig. 2:**
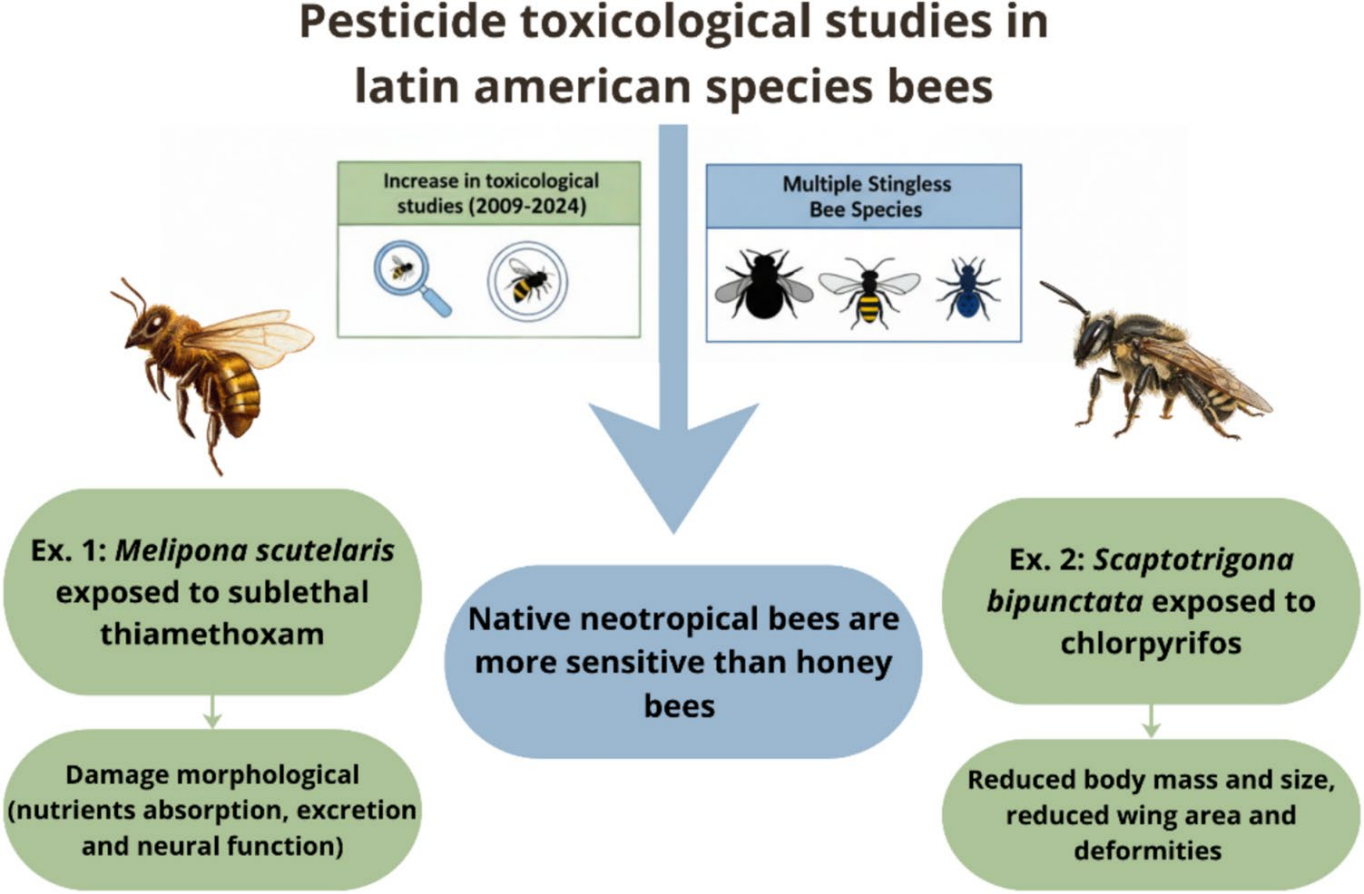
Sublethal effects of pesticides on native stingless bees, indicating morphological damage that may lead to increased mortality rates (Dorneles et al. 2021; Miotello et al. 2022). Source: Authors

Over the last 15 years, toxicological studies in Brazil and elsewhere in Latin America have increased, focusing on acute and sublethal effects of pesticides on honey bees and multiple stingless bee species (Valdovinos Núñez et al. 2009; Freitas & Pinheiro 2010; Pinheiro & Freitas 2010; Lourenço et al. 2012; Barbosa et al. 2015; Tavares et al. 2015; Soares et al. 2015; Lima et al. 2016; Cham et al. 2017; Tomé et al. 2017; Botina et al. 2020; Ribas et al. 2024), revealing that native Neotropical bees can be more sensitive than honey bees to several compounds. For example, in the Brazilian stingless bee *Melipona scutellaris*, exposure to food-ingested sublethal concentration of thiamethoxam (a broad spectrum neonicotinoid) was associated with morphological damage to internal organs related to nutrient absorption, excretion, and neural function—without directly testing those functions (Miotelo et al. 2022). Similarly, *Scaptotrigona bipunctata* workers exposed to chlorpyrifos (an organophosphorus insecticide/acaricide), through contamination of the larval diet, showed reduced body mass and size, and ≈28% of emerged adults had reduced wing area and deformities (Dorneles et al. 2021). These results indicate that stingless bees may have reduced survival chances when exposed to different pesticides (Fig. 2).

Reflecting accumulating evidence of deleterious effects on pollinators—especially neonicotinoids—the Instituto Brasileiro do Meio Ambiente e dos Recursos Naturais Renováveis—IBAMA (Brazilian Institute of Environment and Renewable Natural Resources) reviewed its pesticide evaluation processes for pollinators. In 2017, it implemented a risk analysis standard that explicitly incorporates different exposure routes (direct and indirect) and sublethal effects (Cham et al. 2017, 2020).

As in other countries, the IBAMA relies on tests with *Apis mellifera* to conduct environmental risk assessments of pesticides on pollinators. This species has been widely used as a surrogate due to its broad geographic distribution, well-known biology, and ease of laboratory maintenance. However, uncertainties remain regarding whether *A. mellifera* is the most suitable indicator species to ensure protection of native pollinators. In 2017, the Workshop on Pesticide Exposure Assessment Paradigm for Non-Apis Bees (United States; with Brazilian participation) concluded that, although current risk-assessment procedures for honey bees are largely conservative, non-Apis bees may experience exposure routes unique to this group and requiring further investigation (Boyle et al. 2019). As a follow-up, IBAMA established a Technical Working Group with representatives from academia, EMBRAPA, the private sector, and the MMA to discuss risk-assessment approaches reflecting Brazilian conditions. The GTT recommended assessing the feasibility of incorporating one or a few native bee species into the framework to better represent Brazil’s pollinator diversity. Consequently, in 2018, a list of candidate native bee species suitable for pesticide risk assessment was published (Pires et al. 2018). In addition, recent work highlights the lack of testing protocols adapted for native stingless bees and ongoing efforts to adapt OECD protocols (e.g., Botina et al. 2020; Dorigo et al. 2019).

The persistence and expansion of legal permits for highly toxic pesticides in Brazil, even for active ingredients banned or restricted in parts of Europe or North America, about 30% of agrochemicals used in Brazil have been banned in the European Union (Bombardi 2017), combined with frequent human poisonings associated with agrochemical use in rural areas and rising reports of honey bee colony losses, have heightened public concern among scientists, beekeepers, health professionals, and conservationists (Rocha Franco & Pelaez 2016; Vasconcelos 2018; Vieira 2019). Although Bombardi (2017) noted that the Brazilian Pesticides Law - Law 7.802/1989 prioritizes re‑evaluation under strong evidence of human carcinogenic, mutagenic, and teratogenic risks (in contrast to the EU’s periodic review scheme in Directive 91/414/EEC), environmental re‑evaluations do occur in Brazil when ecological harm is evidenced. For example, in 2012, IBAMA initiated re‑assessments of imidacloprid, thiamethoxam, clothianidin, and fipronil due to accumulating scientific evidence and bee mass‑mortality reports (Cham et al. 2017). On March 31, 2021, IBAMA concluded the environmental reassessment of the neonicotinoid imidacloprid and submitted a Technical Opinion to the Ministério da Agricultura e Pecuária—MAPA (n.d.) (Ministry of Agriculture and Livestock), including use restrictions for specific crops (e.g., exclusion during inflorescence), prohibition in seed‑production crops, limitations for other crops, and labeling warnings such as: “This product is toxic to bees. Non‑target broadcast spraying is not allowed. Do not apply during or immediately before the flowering period”.

Since 1985, the Fundação Oswaldo Cruz—Fiocruz (Oswaldo Cruz Foundation; Fiocruz) has annually published national poison‑exposure statistics via the Sistema Nacional de Informações Tóxico‑Farmacológicas—SINITOX (National System of Toxic‑Pharmacological Information; SINITOX), based on reports from Poison Control and Information Centers. Between 1999 and 2015, major categories included medicines (20,000–35,000 cases/year; 50–107 deaths), venomous animals (snakes, scorpions, spiders, bees; 15,500–27,700; 36–48 deaths), agrochemicals used in agriculture (3300–6300; 97–190 deaths), rodenticides (2800–4400; 20–89 deaths), and addictive drugs (2100–7100; 11–71 deaths). Although agricultural‑agrochemical poisonings ranked third in frequency, they accounted for the largest share of deaths among these categories. Under‑reporting is substantial: the World Health Organization (Pignati et al. 2014 ) estimates approximately 50 unreported cases for each reported case, suggesting ~ 165,000–315,000 agrochemical‑related cases annually in rural areas.

The "Agência Nacional de Vigilância Sanitária" — Anvisa (National Health Surveillance Agency) has monitored pesticide residues in foods since 2001 via the "Programa de Análise de Resíduos de Agrotóxicos em Alimentos—PARA (Pesticide Residue Analysis Program in Food). From 2013 to 2015, 12,051 samples of 25 foods representative of Brazilian diets were analyzed for 232 agrochemicals: 80.3% were compliant (42.0% with no residues; 38.3% below Maximum Residue Limits), and 19.7% were unsatisfactory (18.3% with non‑authorized substances for that crop; 3.0% above limits) (Anvisa 2016).

### Promoting sustainable agricultural solutions

Brazil has a century-long tradition of biological control against agricultural pests (Alves 1998; Parra et al. 2002; Bueno 2009). Risk assessment, importation, and quarantine procedures are formally established by the Instituto Brasileiro do Meio Ambiente e dos Recursos Naturais Renováveis—IBAMA (Brazilian Institute of Environment and Renewable Natural Resources) and the Embrapa with a main quarantine facility for imported biocontrol organisms at Embrapa Meio Ambiente (Embrapa Environment Center) in Jaguariúna, São Paulo, since 1991 (Sá et al. 2016). Overviews of recent progress are available in Parra (2014), Parra & Coelho (2019), and Mascarin et al. (2018). A cost–benefit case study for sugarcane is provided by Renzi et al. (2019).

The expansion of biocontrol in Brazil led to the establishment of the Associação Brasileira das Empresas de Controle Biológico—ABCBio (Brazilian Association of Biological Control Companies; ABCBio) in 2007, which currently includes dozens of companies commercializing hundreds of products. The market experienced rapid growth (e.g., + 70% in 2018), contributing to reduced reliance on conventional agrochemicals (ABCBio 2018). A recent ABCBio survey indicated that 57% of farmers were aware of biocontrol products and 39% were users, often in combination with agrochemicals. Reported adoption approaches ~ 20% for soybean and coffee (historically higher before recent technological packages), ~ 20–40% for sugarcane, beans, apple, and grapes, and > 40% for potato, melon, strawberry, tomato, and vegetables/greens. Farmers cited efficiency (76%) and applicator safety (60%) as main reasons for adoption (Barsari & Claudino 2018). In recent years, some farmers have established on-farm production of parasitoids and microorganisms for mass release as a response to rising costs of imported agrochemicals.

According to ABCBio (ABCBio n.d.), “biological control” refers to the use of organisms or natural substances produced by organisms to prevent, reduce, or eradicate pest and disease outbreaks. Products are commonly grouped as:

1. Macro-organisms (insects, mites, nematodes that parasitize or prey on pests);
2. Microorganisms (bacteria, fungi, viruses that infect pests);
3. Biochemicals (plant/algal extracts, enzymes, hormones that can induce plant resistance); and
4. Semiochemicals (pheromones and related metabolites used for monitoring, mass trapping, and mating disruption).

Regarding “reduced risk” pesticides, the US Environmental Protection Agency (EPA) classifies them by comparative criteria, including (1) low impact on human health; (2) low toxicity to non-target organisms; (3) low potential for groundwater contamination; (4) lower use rates; (5) low resistance potential; and (6) compatibility with Integrated Pest Management — IPM (Barbosa et al. 2015). The “biopesticide” concept is frequently used in a broad sense to include molecules of biological origin as well as living agents. A common misconception is that biological origin implies lower risk; in reality, toxicity depends on chemical structure and exposure conditions, not on natural vs. synthetic origin. Consequently, some biopesticides and reduced risk products can still exert lethal and/or sublethal effects on honeybees and stingless bees, potentially approaching impacts reported for neonicotinoids in certain contexts (Barbosa et al. 2015). Complementarily, recent studies discuss biopesticides and native bees in Brazil and highlight evidence gaps in risk assessment for non-targets (e.g., Catania et al. 2023; Lima et al. 2024). These concerns do not apply uniformly to all forms of biological control (e.g., macro/micro organism releases), which undergo specific regulatory pathways and risk assessment under Brazilian law.

Since 2001, the Ministério da Agricultura e Pecuária—MAPA (Ministry of Agriculture and Livestock; MAPA) has implemented the Programa de Produção Integrada de Alimentos (Integrated Food Production Program), promoting sustainable production across > 60 value chains (grains, tubers, oilseeds, biofuels, vegetables, flowers, fruits, medicinal plants, meat, milk, honey). More recently, MAPA launched the Programa Nacional de Bioinsumos (National Bio-inputs Program) (Decree No. 10.375/2020) to foster the development and use of biological products for pest control and biofertilizers. According to the Censo Agropecuário (IBGE 2018), farms using agrochemicals increased by 23.1% from 2006 to 2017 (1,396,077 to 1,815,361), while 3,230,186 farmers (64%), mostly family farmers, reported not using agrochemicals, likely to reduce costs. Certified organic production increased ~ 400% from 2010 to 2017 (from 5406 to 20,050 farms; MAPA). Since 2013, Brazil has implemented the Política Nacional de Agroecologia e Produção Orgânica—PNAPO (National Policy on Agroecology and Organic Production) (Dias 2018). To meet the goals of the second PLANAPO (2016–2019), the legal framework of the National Bio-inputs Program was launched after ~ 5 years of multi-stakeholder development, establishing standards for biological products used in plant and animal production (Vidal et al. 2021). Further reinforcing these efforts, the federal government instituted the Programa Nacional de Redução de Agrotóxicos—PRONARA (National Program for Pesticide Reduction) by Decree No. 12.538 (Brasil, 2025), integrated into PNAPO, to promote the gradual and continuous reduction of agrochemical use, especially highly hazardous products by incentivizing sustainable practices, strengthening bio inputs, and enhancing intersectoral monitoring and control. PRONARA also emphasizes healthy food systems, the human right to health, and research and innovation in agroecological production.

## Conclusions and recommendations

**Fig. 3:**
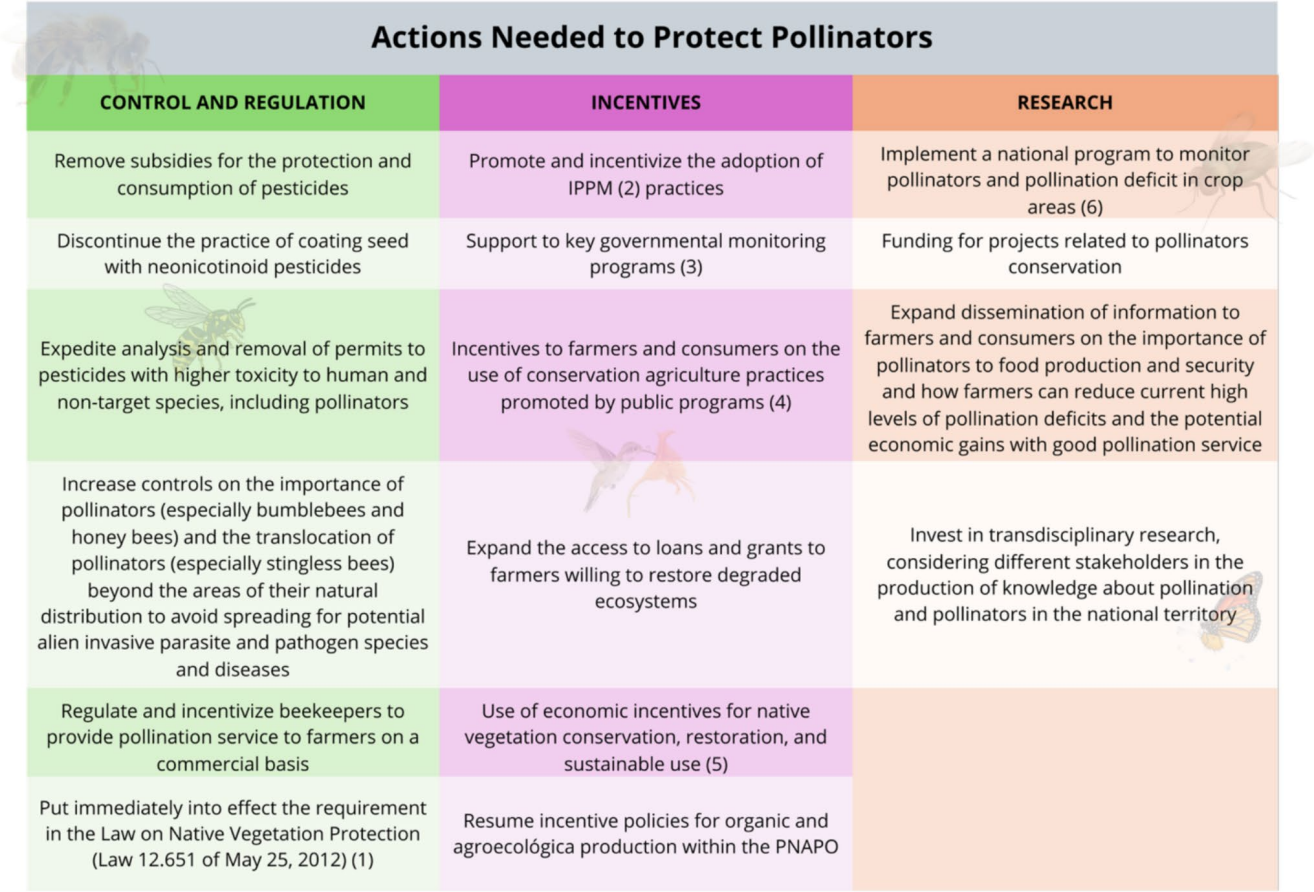
Main aspects that still need to be strengthened to have Pollinators Protection in Brazil. Targets are based on three main aspects: Control and Regulation, Incentives, and Research. (1) Only farmers in compliance with the environmental requirements of the Rural Environmental Registry (CAR) should be eligible for access to public funding to farmers; (2) IPPM—Integrated pest and pollinator management; (3) Such as the National System on Toxicological and Pharmacologic Information—SINITOX (by FIOCRUZ) and the Program of Analysis of Agrochemicals Residues in Food—PARA (by ANVISA); (4) Such as Integrated Production Systems, Low Carbon Emission Agriculture, agroecology, and organic farming as promoted by the National Policy for Agroecology and Organic Production (FAO 2019), winner of the silver award of the Future Policy Award of 2018 for best policies on agroecology and sustainable food systems; (5) An example is chapter 10 of the Law on Native Vegetation Protection (Law 12.651 of May 25, 2012); (6) This national program should be based on the lessons learned in the implementation of the Brazilian Pollinators Initiative as part of the International Initiative to Pollinators’ Conservation and Sustainable Use—IPI and the International Project “Conservation and Management for Pollinators for Sustainable Agriculture, Through an Ecosystem Approach” under the Food and Agriculture Organization of the United Nations (FAO) coordination (MMA/Polinizadores)

Despite the global and national challenges related to pollinator threats, there remains significant scope and a robust legal framework to focus on and invest in promising strategies and plans for pollinator conservation in Brazil. Initiatives such as the National Action Plan for Pollinators Conservation, currently being finalized, and strong research networks like REBIPP and INPOL, which unites many researchers working on pollinators, are crucial. Future endeavors, such as the proposed Regional Action for the Enhanced Protection of Pollinating Insects and Pollination Services in Latin America (Poli-LAC), presently under negotiation, also offer pathways for pollinator protection and sustainable development in the country. While Brazil possesses significant legislation related to biodiversity conservation, there is still a need for more comprehensive and interdisciplinary legislation specifically addressing pollinator conservation (Hipólito et al. 2021). Furthermore, it is essential to implement best practices for pollinator protection and the sustainable use of pollinators across various agricultural production systems. Existing legislation and policies should be enhanced; we outlined key aspects summarized in Fig. 3.

## References

[CR1] Abcbio (2018) Mercado de biodefensivos cresce mais de 70% no Brasil em um ano. Associação Brasileira das Empresas de Controle Biológico – ABCBIO, São Paulo. Available at: http://www.agricultura.gov.br/noticias/feffmercado-de-biodefensivos-cresce-em-mais-de-50-no-brasil.

[CR2] Abcbio (n.d.) Associação Brasileira das Empresas de Controle Biológico – ABCBIO, São Paulo. Available at: http://www.abcbio.org.br/. Accessed: 17 de maio de 2024.

[CR3] Abreu PHB, Alonzo HGA (2014) Trabalho rural e riscos à saúde: uma revisão sobre o “uso seguro” de agrotóxicos no Brasil. Cienc Saude Coletiva 19:4197–4208. 10.1590/1413-812320141910.09342014 10.1590/1413-812320141910.09342014 25272128

[CR4] Aguilar R, Ashworth L, Galetto L, Aizen MA (2006) Plant reproductive susceptibility to habitat fragmentation: review and synthesis through a meta-analysis. Ecol Lett 9:968–980. 10.1111/j.1461-0248.2006.00927.x 10.1111/j.1461-0248.2006.00927.x 16913941

[CR5] Aizen MA, Aguiar S, Biesmeijer JC et al (2019) Global agricultural productivity is threatened by increasing pollinator dependence without a parallel increase in crop diversification. Glob Change Biol 25:3516–3527. 10.1111/gcb.14736 10.1111/gcb.14736 PMC6852307 31293015

[CR6] Alves SB (ed.) (1998) Controle microbiano de insetos. 2.ed. FEALQ, Piracicaba, 1063p.

[CR7] Anvisa (2016) Programa de Análise de Resíduos de Agrotóxicos em Alimentos – PARA. Relatório das Análises de Amostras Monitoradas no Período 2013 a 2015. Agência Nacional de Vigilância Sanitária – ANVISA, Brasília, 246p.

[CR8] Barbosa WF, Smagghe G, Guedes RNC (2015) Pesticides and reduced-risk insecticides, native bees and pantropical stingless bees: pitfalls and perspectives: pesticides and reduced-risk insecticides, native bees and pantropical stingless bees. Pest Manag Sci 71:1049–1053. 10.1002/ps.4025 25892651 10.1002/ps.4025

[CR9] Barsari AP, & Claudino M (2018) Biodefensivos: mercado e percepção do Produtor Brasileiro. Analysis, São Paulo (Associação Brasileira das Empresas de Controle Biológico – ABCBIO). Available at: http://www.abcbio.org.br/conteudo/publicacoes/.

[CR10] Benjamin EF, Reilly RJ, Winfree R (2014) Pollinator body size mediates the scale at which land use drives crop pollination services. J Appl Ecol 51:440–449. 10.1111/1365-2664.12198

[CR11] Bernardes RC, Botina LL, Araújo R, Guedes RNC, Martins GF, Lima MAP (2022) Artificial intelligence-aided meta-analysis of toxicological assessment of agrochemicals in bees. Front Ecol Evol 10:845608. 10.3389/fevo.2022.845608

[CR12] Biddinger DJ, Rajotte EG (2015) Integrated pest and pollinator management—adding a new dimension to an accepted paradigm. Curr Opin Insect Sci 10:204–209. 10.1016/j.cois.2015.05.012 29588010 10.1016/j.cois.2015.05.012

[CR13] Biesmeijer JC, Roberts SPM, Reemer M, Ohlemuller R, Edwards M, Peeters T, Schaffers AP, Potts SG, Kleukers R, Thomas CD, Settele J, Kunin WE (2006) Parallel declines in pollinators and insect-pollinated plants in Britain and the Netherlands. Science 313(5785):351–354. 10.1126/science.1127863 16857940 10.1126/science.1127863

[CR14] Blaauw BR, Isaacs R (2014) Flower plantings increase wild bee abundance and the pollination services provided to a pollination-dependent crop. J Appl Ecol 51:890–898. 10.1111/1365-2664.12257

[CR15] Bombardi LM (2017) Geografia do uso de agrotóxicos no Brasil e conexões com a União Europeia. Laboratório de Geografia Agrária – FFLCH-USP, São Paulo, 296p.

[CR16] Botina JP, Nocelli RCF, Monquero PA, Simões ZLP (2020) Toxicological assessments of agrochemical effects on stingless bees (Apidae, Meliponini): a systematic review. Chemosphere 240:124888 10.1016/j.mex.2020.100906 PMC7225395 32426248

[CR17] Botina LL, Barbosa WF, Martins GF (2024) Toxicological assessments of agrochemicals in stingless bees in Brazil: a systematic review. Neotrop Entomol 53(3):480–489. 10.1007/s13744-024-01132-x 38358646 10.1007/s13744-024-01132-x

[CR18] Boyle NK, Pitts-Singer TL, Abbott J, Alix A, Cox-Foster DL, Hinarejos S, Lehmann DM, Morandin L, O’Neill B, Raine NE, Singh R (2019) Workshop on pesticide exposure assessment paradigm for non-apis bees: foundation and summaries. Environ Entomol 48(1):4–11. 10.1093/ee/nvy103 30508116 10.1093/ee/nvy103 PMC8381227

[CR19] Brasil (2025) Decreto nº 12.538, de 30 de junho de 2025. Institui o Programa Nacional de Redução de Agrotóxicos. Diário Oficial da União, Brasília, DF, 1 jul. 2025. Seção 1, p. 1.

[CR20] Buchmann SL, & Nabhan GP (1996) The forgotten pollinators. Island Press, Washington (DC), 292p.

[CR21] Bueno VHP (ed.) (2009) Controle biológico de pragas: produção massal e controle de qualidade. 2.ed. Editora da Universidade Federal de Lavras (UFLA), Lavras (MG), 430p.

[CR22] Butchart SHM, Walpole M, Collen B et al (2010) Global biodiversity: indicators of recent declines. Science 328:1164–1168. 10.1126/science.1187512 20430971 10.1126/science.1187512

[CR23] Byrne A, Fitzpatrick Ú (2009) Bee conservation policy at the global, regional and national levels. Apidologie 40:194–210. 10.1051/apido/2009017

[CR24] Calderone NW (2012) Insect pollinated crops, insect pollinators and US agriculture: trend analysis of aggregate data for the period 1992–2009. PLoS One 7:e37235. 10.1371/journal.pone.0037235 22629374 10.1371/journal.pone.0037235 PMC3358326

[CR25] Câmara Dos Deputados (2013) Audiência Pública da Comissão de Meio Ambiente sobre “Mortandade disseminada das abelhas devido ao uso de agrotóxicos”, em 4 de julho de 2013, apresentações realizadas. Available at: <http://www2.camara.leg.br/atividade-legislativa/comissoes/comissoes-permanentes/cmads/audiencias-publicas/audiencia-publica-2013/4-7-2013-mortandade-disseminada-das-abelhas-devido-ao-uso-deagrotoxicos/apresentacoes/>.

[CR26] Campanhola C, Rodrigues GS, Dias BFS (1998) Agricultural biological diversity. Cienc Cult Sao Paulo 50(1):10–13

[CR27] Carvalheiro LG, Seymour CL, Veldtman R, Nicolson SW (2010) Pollination services decline with distance from natural habitat even in biodiversity-rich areas. J Appl Ecol 47:810–820

[CR28] Castilhos D (2018) Desaparecimento e morte de abelhas no Brasil, registrados no aplicativo Bee Alert. Tese de Doutorado, Universidade Federal Rural do Semi-Árido (UFERSA), Departamento de Ciências Animais, Programa de Pós-graduação em Ciência Animal, Mossoró (RN), 163p.

[CR29] Castilhos D, Bergamo GC, Gramacho KP, Gonçalves LS (2019) Bee colony losses in Brazil: a 5-year online survey. Apidologie 50(3):263–272

[CR30] Catania R, Lima MAP, Potrich M, Sgolastra F, Zappalà L, Mazzeo G (2023) Are botanical biopesticides safe for bees (Hymenoptera, Apoidea)? Insects 14(3):247. 10.3390/insects14030247 36975932 10.3390/insects14030247 PMC10053700

[CR31] CBD - Convention on Biological Diversity (2022). Kunming-Montreal Global Biodiversity Framework: Decision adopted by the Conference of the Parties to the Convention on Biological Diversity (CBD/COP/DEC/15/4). https://www.cbd.int/doc/decisions/cop-15/cop-15-dec-04-en.pdf

[CR32] CBD Secretariat - Convention on Biological Diversity (2016a) Strategic actions to enhance the implementation of the Strategic Plan for Biodiversity 2011–2020 and the achievement of the Aichi Biodiversity Targets, including with respect to mainstreaming and the integration of biodiversity within and across sectors. Convention on Biological Diversity, Montreal, CBD/COP/DEC/XIII/3, 19p.

[CR33] CBD Secretariat - Convention on Biological Diversity (2016b) Implications of the IPBES assessment on pollinators, pollination and food production for the work of the Convention. Convention on Biological Diversity, Montreal, CBD/COP/DEC/XIII/15, 5p.

[CR34] CBD Secretariat - Convention on Biological Diversity (2018) Conservation and sustainable use of pollinators. Convention on Biological Diversity, Montreal, CBD/COP/DEC/XIV/6, 16p. (Annex I: Updated Plan of Action 2018–2030 for the International Initiative on the Conservation and Sustainable Use of Pollinators).

[CR35] CHAM K de O, Rebelo RM, Oliveira R de P, Ferro AA, de C. Viana-Silva FE,de O. Borges L, Saretto COSD, Tonelli CAM, & Macedo TC (2020) Manual de avaliação de risco ambiental de agrotóxicos para abelhas. Ibama/Diqua, Brasília, 114 p

[CR36] Cham KO, Nocelli RCF, Borges LO et al (2019) Pesticide exposure assessment paradigm for stingless bees. Environ Entomol 48:36–48. 10.1093/ee/nvy137 30508180 10.1093/ee/nvy137

[CR37] Cham K, Tonelli C, Borges L, & Silva FV (2017) Atual cenário da avaliação de risco de agrotóxicos para polinizadores no Brasil, p. 67–74. In: CGEE, Importância dos polinizadores na produção de alimentos e na segurança alimentar global. Centro de Gestão e Estudos Estratégicos, Brasília, 123p.

[CR38] Coutinho JGE, Hipólito J, Santos RLS et al (2021) Landscape structure is a major driver of bee functional diversity in crops. Front Ecol Evol 9:624835. 10.3389/fevo.2021.624835

[CR39] Cunha HJ, & Landeiro MCPP (2012) Polinizadores e Políticas Públicas, p. 435–461. In: Imperatriz-Fonseca, V. L., D. A. L. Canos, D. A. Alves & A. M. Saraiva (orgs.). Polinização no Brasil: Contribuição e Perspectivas para a Biodiversidade, Uso Sustentável, Conservação e Serviços Ambientais. Editora da Universidade de São Paulo - EdUSP, São Paulo, 485p.

[CR40] Dainese M, Martin EA, Aizen MA, Albrecht M, Bartomeus I, Bommarco R et al (2019) A global synthesis reveals biodiversity-mediated benefits for crop production. Sci Adv 5(10):eaax0121. 10.1126/sciadv.aax0121 31663019 10.1126/sciadv.aax0121 PMC6795509

[CR41] De Benedicto SC, Castro JPS, Sugahara CR, Silva Filho CF (2019) The Brazilian agribusiness and the consequences of the intensive use of agrochemicals. Int J Innov Educ Res 7(1):170–186

[CR42] Degrandi-Hoffman G, Chen Y (2015) Nutrition, immunity and viral infections in honey bees. Curr Opin Insect Sci 10:170–176. 10.1016/j.cois.2015.05.007 29588005 10.1016/j.cois.2015.05.007

[CR43] de Moraes RF (2022) Constructing a transnational crime: pesticide smuggling in Brazil. Crime Law and Social Change 78(4):379–404. 10.1007/s10611-022-10026-1

[CR44] Dias De Freitas C, Oki Y, Resende FM et al (2022) Impacts of pests and diseases on the decline of managed bees in Brazil: a beekeeper perspective. J Apic Res 1:1–14. 10.1080/00218839.2022.2099188

[CR45] Dias BFS (coord.) (2006) Bibliografia brasileira de polinização e polinizadores. 2. ed. Ministério do Meio Ambiente, Secretaria de Biodiversidade e Florestas (série Biodiversidade 16), Brasília, 224p.

[CR46] Dias BFS (2018) Second International Symposium on Agroecology: Scaling up Agroecology to achieve the Sustainable Development Goals, 3–5 April 2018 Rome. Chair’s Summary. Food and Agriculture Organization of the United Nations – FAO, Rome, 6p.

[CR47] Dias BFS (2021a) Degradação da Biodiversidade e as Metas de Aichi no Mundo e no Brasil: um balanço dos avanços e das perspectivas. BioDiverso, Porto Alegre 1:22–44

[CR48] Dias BFS (2021b) Estratégia de uso e conservação da biodiversidade para adaptação da agropecuária brasileira às mudanças do clima, p. 108–111, 182. In: SOTTA, E. D., F. G. Sampaio, K. Marzall & W. G. da Silva (orgs.). Estratégias de adaptação às mudanças do clima dos sistemas agropecuários brasileiros. Ministério da Agricultura, Pecuária e Abastecimento - MAPA, Brasília, 187p.

[CR49] Dias BFS, Raw A, & Imperatriz-Fonseca V (coords.) (1999) The São Paulo declaration on pollinators: Report of the International Workshop on the Conservation and Sustainable use of Pollinators in Agriculture, with Emphasis on Bees. Ministério do Meio Ambiente, Brasília, 51p. Available at: http://www.mma.gov.br/estruturas/chm/_arquivos/pollinas.pdf.

[CR50] Dicks LV, Viana B, Bommarco R et al (2016) Ten policies for pollinators. Science 354:975–976. 10.1126/science.aai9226 27884996 10.1126/science.aai9226

[CR51] Dorigo AS, Rosa-Fontana A, Soares-Lima HM, Galaschi-Teixeira JS, Nocelli RCF, Malaspina O (2019) In vitro larval rearing protocol for the stingless bee species *Melipona scutellaris* for toxicological studies. PLoS One 14(3):e0213109. 10.1371/journal.pone.0213109 30893338 10.1371/journal.pone.0213109 PMC6426188

[CR52] Dorneles AL, de S. Souza Rosa A, Blochtein B (2017) Toxicity of organophosphorus pesticides to the stingless bees *Scaptotrigona bipunctata* and *Tetragonisca fiebrigi*. Apidologie 48:612–620. 10.1007/s13592-017-0502-x

[CR53] Dorneles AL, de S. Rosa-Fontana A, dos Santos CF, Blochtein B (2021) Larvae of stingless bee *Scaptotrigona bipunctata* exposed to organophosphorus pesticide develop into lighter, smaller and deformed adult workers. Environ Pollut 272:116414. 10.1016/j.envpol.2020.116414 33445151 10.1016/j.envpol.2020.116414

[CR54] Dos Santos CF, Otesbelgue A, Blochtein B (2018) The dilemma of agricultural pollination in Brazil: beekeeping growth and insecticide use. PLoS One 13:e0200286. 10.1371/journal.pone.0200286 29979763 10.1371/journal.pone.0200286 PMC6034858

[CR55] Eardley C, Roth D, Clarke J, Buchmann S, Gemmill B (2006) Pollinators and Pollination: A resource book for policy and practice, vol xv+77p. African Pollinator Initiative, Pretoria

[CR56] Ellis JD, Evans JD, Pettis J (2010) Colony losses, managed colony population decline, and colony collapse disorder in the United States. J Apic Res 49(1):134–136

[CR57] FAO (2019) Scaling up agroecology to achieve the sustainable development goals. Proceedings of the second FAO international symposium, 3-5 April 2018. Food and Agriculture Organization of the United Nations – FAO, Rome, 412p. [chair: Braulio Ferreira de Souza Dias]

[CR58] FAO (2020a) Global Forest Resources Assessment 2020: Main report. Food and Agriculture Organization of the United Nations - FAO, Rome, p 186. 10.4060/ca9825en

[CR59] FAO (2020b) Towards Sustainable Crop Pollination Services Measures at Field, Farm and Landscape Scales. B. Gemmill-Herren, N. Azzu, A. Bicksler & A. Guidotti (eds.). Food and Agriculture Organization of the United Nations (Commission on Genetic Resources for Food and Agriculture), Rome, 194p

[CR60] FAO - FAOSTAT (2022) Food and Agriculture Organization of the United Nations (FAO), 2022, https://www.fao.org/faostat/

[CR61] Ferreira PA, Boscolo D, Carvalheiro LG et al (2015) Responses of bees to habitat loss in fragmented landscapes of Brazilian Atlantic Rainforest. Landsc Ecol 30:2067–2078. 10.1007/s10980-015-0231-3

[CR62] Fleming TH, Geiselman C, Kress WJ (2009) The evolution of bat pollination: a phylogenetic perspective. Ann Bot 104:1017–1043. 10.1093/aob/mcp197 19789175 10.1093/aob/mcp197 PMC2766192

[CR63] Freitas BM, & Bomfim IGA (2017) A necessidade de uma convivência harmônica da agricultura com os polinizadores, p. 39-50. In: CGEE, Importância dos polinizadores na produção de alimentos e na segurança alimentar global. Centro de Gestão e Estudos Estratégicos, Brasília, 123p

[CR64] Freitas BM, Pinheiro JN (2010) Efeitos sub-letais dos pesticidas agrícolas e seus impactos no manejo de polinizadores dos agroecossistemas brasileiros. Oecol Aust 14(1):282–298

[CR65] Freitas de PVDX, Ribeiro FM, de Almeida EM et al (2017) Declínio populacional das abelhas polinizadoras: Revisão. Pubvet 11(1):1–10

[CR66] Gallai N, Salles JM, Settele J, Vaissière BE (2009) Economic valuation of the vulnerability of world agriculture confronted with pollinator decline. Ecol Econ 68(3):810–821. 10.1016/j.ecolecon.2008.06.014

[CR67] Gemmill-Herren B (ed.) (2016) Pollination Services to Agriculture: Sustaining and enhancing a key ecosystem service. FAO, Rome & Earthscan/Routledge, London, xvii+283p.

[CR68] Giannini TC, BoffS Cordeiro GD, Cartolano EA, Veiga AK, Imperatriz-Fonseca VL, Saraiva AM (2015) Crop pollinators in Brazil: a review of reported interactions. Apidologie 46(2):209–223

[CR69] Giannini TC, Speranza CFFG, de Oliveira JB, de SM, de Castro ACBCM, de Oliveira MCM, de Paiva R L, de Faria SDG, Silva ENC, da Silva JAO, & Imperatriz-Fonseca VL (2020) Demand and supply of animal pollination in the Brazilian agricultural landscape: An urgent need for improved land-use planning. Agric Ecosyst Environ 297:106927. 10.1016/j.agee.2020.106927

[CR70] Goulson D (2013) An overview of the environmental risks posed by neonicotinoid insecticides. J Appl Ecol 50:977–987

[CR71] Goulson D, Nicholls E, Botias C, Rotheray EL (2015) Bee declines driven by combined stress from parasites, pesticides, and lack of flowers. Science 347:1255957. 10.1126/science.1255957 25721506 10.1126/science.1255957

[CR72] Gray A, Adjlane N, Arab A et al (2023) Honey bee colony loss rates in 37 countries using the COLOSS survey for winter 2019–2020: the combined effects of operation size, migration and queen replacement. J Apic Res 62:204–210. 10.1080/00218839.2022.2113329

[CR73] Hall DM, Steiner R (2019) Insect pollinator conservation policy innovations at subnational levels: lessons for lawmakers. Environ Sci Policy 93:118–128. 10.1016/j.envsci.2018.12.026

[CR74] Hipólito J, Boscolo D, Viana BF (2018) Landscape and crop management strategies to conserve pollination services and increase yields in tropical coffee farms. Agric Ecosyst Environ 256:218–225. 10.1016/j.agee.2017.09.038

[CR75] Hipólito J, Coutinho J, Mahlmann T, Santana TBR, Magnusson WE (2021) Legislation and pollination: recommendations for policymakers and scientists. Perspect Ecol Conserv 19(1):1–9. 10.1016/j.pecon.2021.01.003

[CR76] Ibama (2020) Paineis de Informações de Agrotóxicos: Painel comercialização. Available at: http://www.ibama.gov.br/agrotoxicos/paineis-de-informacoes-de-agrotoxicos#Painel-comercializacao. Accessed: 17 de maio de 2024

[CR77] Ibge (2018) Censo Agropecuário 2017: Resultados preliminares. Instituto Brasileiro de Geografia e Estatística, Rio de Janeiro, p 108

[CR78] Icmbio (2018) Livro Vermelho da Fauna Brasileira Ameaçada de Extinção, vol 7. Instituto Chico Mendes de Conservação da Biodiversidade, Brasília

[CR79] Imperatriz-Fonseca V, Canos DAL, Alves DA, & Saraiva AM (orgs.) (2012) Polinizadores no Brasil: Contribuição e Perspectivas para a Biodiversidade, Uso Sustentável, Conservação e Serviços Ambientais. Editora da Universidade de São Paulo, São Paulo, 488 p

[CR80] Imperatriz-Fonseca VL, Freitas BM, Saraiva AM, & Dias BFS (2004) The Brazilian Pollinators Initiative: challenges and opportunities, p. 56-62. In: Proceedings of the 8th IBRA International Conference on Tropical Bees and VI Encontro sobre Abelhas. Universidade de São Paulo, Ribeirão Preto (SP)

[CR81] Imperatriz-Fonseca VL, Alves DA, Assad AL, Blochtein B, Freitas B, & Dias BFS (2019) Polinizadores, Polinização e Produção de Alimentos no Brasil. Mensagem Doce (APACAME), São Paulo, 151: 28-30

[CR82] Ipbes (2016a) Summary for Policymakers of the Assessment Report of the Intergovernmental Science-Policy Platform on Biodiversity and Ecosystem Services (IPBES) on Pollinators, Pollination and Food Production. Intergovernmental Science-Policy Platform on Biodiversity and Ecosystem Services Secretariat, Bonn, 40p

[CR83] Ipbes (2016b) The assessment report of the Intergovernmental Science-Policy Platform on Biodiversity and Ecosystem Services on pollinators, pollination and food production. S. G. Potts, V. L. Imperatriz-Fonseca & H. T. Ngo (eds.). Secretariat of the Intergovernmental Science-Policy Platform on Biodiversity and Ecosystem Services, Bonn, 552p

[CR84] IUCN (2019) Guidelines for Recognizing and Reporting Other Effective Area-based Conservation Measures. International Union for Nature Conservation (IUCN)/World Commission on Protected Areas, Gland (Switzerland), 46p

[CR85] Joly CA, Scarano FR, Seixas CS et al (2019) 1o Diagnóstico Brasileiro de Biodiversidade & Serviços Ecossistêmicos. Editora Cubo, São Carlos

[CR86] Kennedy CM, Lonsdorf EV, Neel MC, Williams NM, Waterhouse G, Winfree R, Ricketts TH (2013) A global quantitative synthesis of local and landscape effects on native bee communities. Ecol Lett 16(5):584–599 23489285 10.1111/ele.12082

[CR87] Kevan, P. & V. L. Imperatriz Fonseca (eds.). (2002). Pollinating Bees - The Conservation Link Between Agriculture and Nature. Ministry of the Environment, Brasilia 313p. [reprinted in 2006].

[CR88] Klein A-M, Freitas BM, Bomfim IGA, Boreux V, Fornoff F, Oliveira MO (2020) A Polinização Agrícola por Insetos no Brasil: Um Guia para Fazendeiros, Agricultores, Extensionistas, Políticos e Conservacionistas. Universitats Bibliothek Freiburg, FreiDok plus, p 162

[CR89] Landers JN (2005) PLANTIO DIRETO: Módulo 1 - Histórico, características e benefícios do Plantio Direto. Associação Brasileira de Educação Agrícola Superior ABEASUniversidade de Brasília - UnBFaculdade de Agronomia e Medicina Veterinária – FAV, Brasília, p 113

[CR90] Laurent, M., P. Hendrikx, M. Ribiere-Chabert & M.-P. Chauzat. (2015). A pan‑European epidemiological study on honeybee colony losses 2012‑2014. Available at: http://ec.europa.eu/food/animals/live_animals/bees/docs/bee-report_2012_2014_en.pdf. 10.1371/journal.pone.0172591 PMC5344352 28278255

[CR91] Lewinsohn TM, Agostini K, Lucci Freitas AV, Melo AS (2022) Insect decline in Brazil: an appraisal of current evidence. Biol Lett 18:20220219. 10.1098/rsbl.2022.0219 10.1098/rsbl.2022.0219 PMC9399695 36000221

[CR92] Lima MAP, Martins GF, Oliveira EE, Guedes RNC (2016) Agrochemical-induced stress in stingless bees: peculiarities, underlying basis, and challenges. J Comp Physiol A 202:733–747. 10.1007/s00359-016-1110-3 10.1007/s00359-016-1110-3 27401560

[CR93] Lima RCA, Harfuch L, Palauro GR (2020) Plano ABC: Evidências do período 2010-2020 e propostas para uma nova fase 2021-2030. Agroicone, São Paulo, p 145

[CR94] Lima MAP, Bernardes RC, Ferreira LMN, Catania R, Mazzeo G (2024) Non-target effects of biopesticides on stingless bees (Apidae, Meliponini): recent trends and insights. Curr Opin Environ Sci Health 42:100580. 10.1016/j.coesh.2024.100580

[CR95] Londres, F. (2011). Agrotóxicos no Brasil: um guia para ação em defesa da vida. Articulação Nacional de Agroecologia, Rio de Janeiro, 191p.

[CR96] Lopes AV, Porto RG, Cruz-Neto O et al (2021) Neglected diversity of crop pollinators: lessons from the world’s largest tropical country. Perspect Ecol Conserv 19:500–504. 10.1016/j.pecon.2021.06.004

[CR97] Lourencetti APS, Azevedo P, Miotelo L, Malaspina O, Nocelli RCF (2023a) Surrogate species in pesticide risk assessments: toxicological data of three stingless bees species. Environ Pollut 318:120842. 10.1016/j.envpol.2022.120842 36509344 10.1016/j.envpol.2022.120842

[CR98] Lourencetti APS, Azevedo P, Miotelo L, Malaspina O, Nocelli RCF (2023b) Reply to the letter to the editor regarding the article Lourencetti et al. (2023). Environ Pollut 330:121785 37196839 10.1016/j.envpol.2023.121785

[CR99] Lourenço CT, Carvalho SM, Malaspina O, Nocelli RCF (2012) Oral toxicity of Fipronil insecticide against the stingless bee *Melipona scutellaris* (Latreille, 1811). Bull Environ Contam Toxicol 89(4):921–924 22886451 10.1007/s00128-012-0773-x

[CR100] Mader, E., M. Shepherd, M. Vaughan, S. Black & G. Lebughn. (2011). Attracting Native Pollinators: Protecting North America’s Bees and Butterflies. Storey Publishing (Xerxes Society Guide), North Adams (Massachusetts), 371p.

[CR101] Mascarin GM, Lopes RB, Delalibera I Jr., Fernandes EKK (2018) Current status and perspectives of fungal entomopathogens used for microbial control of arthropod pests in Brazil. J Invertebr Pathol 165:46–53 10.1016/j.jip.2018.01.001 29339191

[CR102] Menz MH, Phillips RD, Winfree R, Kremen C, Aizen MA, Johnson SD, Dixon KW (2011) Reconnecting plants and pollinators: challenges in the restoration of pollination mutualisms. Trends Plant Sci 16(1):4–12. 10.1016/j.tplants.2010.09.006 20980193 10.1016/j.tplants.2010.09.006

[CR103] Merotto A, Gazziero DLP, Oliveira MC et al (2022) Herbicide use history and perspective in South America. Adv Weed Sci 40:e020220050. 10.51694/AdvWeedSci/2022;40:seventy-five010

[CR104] Ministério da Agricultura, Pecuária e Abastecimento (MAPA). (2009). Produção integrada no Brasil: agropecuária sustentável alimentos seguros. Ministério da Agricultura, Pecuária e Abastecimento. Secretária de Desenvolvimento Agropecuário e Cooperativismo, Brasília, 1008p. + 1 CD-ROM. Available at: http://www.agricultura.gov.br/assuntos/sustentabilidade/producao-integrada/documentos-producao-integrada/producao-integrada-no-brasil.pdf.

[CR105] Ministério da Agricultura, Pecuária e Abastecimento (MAPA). (2021). ABC+ Plano setorial para adaptação à mudança do clima e baixa emissão de carbono na agropecuária com vistas ao desenvolvimento sustentável (2020-2030): Visão estratégica para um novo ciclo. Ministério da Agricultura, Pecuária e Abastecimento – MAPA/ Secretaria de Inovação, Desenvolvimento Rural e Irrigação, Brasília, 28p.

[CR106] Ministério da Agricultura, Pecuária e Abastecimento (MAPA). (n.d.). Produção integrada no Brasil: agropecuária sustentável alimentos seguros. Ministério da Agricultura, Pecuária e Abastecimento. Secretária de Desenvolvimento Agropecuário e Cooperativismo, Brasília. Available at: http://www.agricultura.gov.br/assuntos/sustentabilidade/producao-integrada and http://www.agricultura.gov.br/assuntos/sustentabilidade/producao-integrada/normas-tecnicas. Accessed: 17 de maio de 2024.

[CR107] Ministério do Meio Ambiente (MMA). (n.d.). Publicações sobre Polinizadores. Ministério do Meio Ambiente, Brasília: portal antigo online. Available at: https://antigo.mma.gov.br/publicacoes/biodiversidade/category/57-polinizadores.html. Accessed: 17 de maio de 2024.

[CR108] Miotelo L, dos Mendes Reis AL, Malaquias JB, Malaspina O, Nocelli RCF (2021) *Apis mellifera* and *Melipona scutellaris* exhibit differential sensitivity to thiamethoxam. Environ Pollut 268:115770. 10.1016/j.envpol.2020.115770 33045589 10.1016/j.envpol.2020.115770

[CR109] Miotello L, Souza A, Roat AS, Pachu JL, Bruno JMA, Malaspina O, Nocelli RCF (2022) A food-ingested sublethal concentration of thiamethoxam has harmful effects on the stingless bee *Melipona scutellaris*. Chemosphere 288:132461. 10.1016/j.chemosphere.2021.132461 34624342 10.1016/j.chemosphere.2021.132461

[CR110] Mitidiero Junior, M. A. & Y. Goldfarb. (2021). O AGRO não é Tech, o AGRO não é Pop e muito menos Tudo. Friedrich-Ebert-Stiftung (FES) Brasil & Associação Brasileira de Reforma Agrária (ABRA), São Paulo, 40p.

[CR111] Morandin LA, Kremen C (2013) Hedgerow restoration promotes pollinator populations and exports native bees to adjacent fields. Ecol Appl 23:829–839. 10.1890/12-1051.1 23865233 10.1890/12-1051.1

[CR112] Nieto A, Roberts SPM, Kemp J, Rasmont P, Kuhlmann M, García Criado M, Biesmeijer JC, Bogusch P, Dathe HH, la De Rúa P, De Meulemeester T, Dehon M, Dewulf A, Ortiz-Sánchez FJ, Lhomme P, Pauly A, Potts SG, Praz C, Quaranta M, Radchenko VG, Scheuchl E, Smit J, Straka J, Terzo M, Tomozii B, Wood TJ, Michez D (2014) European Red List of Bees. Publications Office of the European Union. 10.2779/77003

[CR113] Oliveira CM, Auad AM, Mendes SM, Frizzas MR (2014) Crop losses and the economic impact of insect pests on Brazilian agriculture. Crop Prot 56:50–54

[CR114] Oliveira W, Colares LF, Porto RG et al (2024) Food plants in Brazil: origin, economic value of pollination and pollinator shortage risk. Sci Total Environ 912:169147. 10.1016/j.scitotenv.2023.169147 38065486 10.1016/j.scitotenv.2023.169147

[CR115] Parra JRP (2014) Biological control in Brazil an overview. Sci Agric 71:420–429

[CR116] Parra JRP, Coelho A Jr. (2019) Applied biological control in Brazil: from laboratory assays to field application. J Insect Sci 19(2):1–6 10.1093/jisesa/iey112 PMC6403475 30822777

[CR117] PARRA, J. R. P., P. S. M. Botelho, B. S. Corrêa Ferreira & J. M. S. BENTO (eds.). (2002). Controle biológico no Brasil: parasitóides e predadores. Manole, São Paulo, 609p.

[CR118] Paschoal, A. D. (1979). Pragas, praguicidas e a crise ambiental: problemas e soluções. Fundaçåo Getúlio Vargas, Rio de Janeiro, 102p. [2ª edition in 2019: Pragas, Agrotóxicos e a crise ambiente: Problemas e soluções. Expressão Popular, São Paulo, 181p.].

[CR119] Pettis JS, Lichtenberg EM, Andree M, Stitzinger J, Rose R, Van Engelsdorp D (2013) Crop pollination exposes honey bees to pesticides which alters their susceptibility to the gut pathogen *Nosema ceranae*. PLoS One 8:e70182. 10.1371/journal.pone.0070182 10.1371/journal.pone.0070182 PMC3722151 23894612

[CR120] Pignati W, Oliveira NP, Silva AMCD (2014) Vigilância aos agrotóxicos: quantificação do uso e previsão de impactos na saúde-trabalho-ambiente para os municípios brasileiros. Ciênc saúde coletiva 19:4669–4678. 10.1590/1413-812320141912.12762014 10.1590/1413-812320141912.12762014 25388175

[CR121] Pimentel D (1986) Agroecology and economics. In: Kogan M (ed) Ecological Theory and Integrated Pest Management Practices. Wiley, New York, pp 299–319

[CR122] Pinheiro JN, Freitas BM (2010) Efeitos letais dos pesticidas agrícolas sobre polinizadores e perspectivas de manejo para os agroecossistemas brasileiros. Oecol Aust 14(1):266–281

[CR123] Pires CSS, Pereira FM, Lopes MTR, Nocelli RCF, Malaspina O, Pettis JS (2016) Enfraquecimento e perda de colônias de abelhas no Brasil: há casos de CCD? Pesq Agropec Bras 51(5):422–442

[CR124] Pires CSS, Torezani KRS, Cham KO, Viana-Silva FEC, Borges LO, Tonelli CAM, Saretto COSD, Nocelli RCF, Malaspina O, Cione AP et al (2018) Seleção de espécies de abelhas nativas para avaliação de risco de agrotóxicos. 1. ed. B: Ibama, Brasília, DF, Brazil. 84 p. http://ibama.gov.br/component/phocadownload/file/4667-selecao-de-­especies-de-abelhas-nativas-para-avaliacao-de-risco-de-agrotoxicos

[CR125] PNAPO. (n.d.). Decreto nº 7.794/2012. Available at: http://www.planalto.gov.br/ccivil_03/_ato2011-2014/2012/decreto/d7794.htm. Accessed: 17 de maio de 2024.

[CR126] Porto RG, De Almeida RF, Cruz-Neto O, Tabarelli M, Viana BF, Peres CA, Lopes AV (2020) Pollination ecosystem services: A comprehensive review of economic values research funding and policy actions. Food Secur 12(6):1425–1442. 10.1007/s12571-020-01043-w

[CR127] Potts SG, Biesmeijer JC, Kremen C et al (2010) Global pollinator declines: trends, impacts and drivers. Trends Ecol Evol 25:345–353. 10.1016/j.tree.2010.01.007 20188434 10.1016/j.tree.2010.01.007

[CR128] Procópio SDO, Barizon RRM, Pazianotto RAA et al (2024) Impacts of weed resistance to glyphosate on herbicide commercialization in Brazil. Agriculture 14:2315. 10.3390/agriculture14122315

[CR129] Promote Pollinators. (n.d.). Promote Pollinators. Available at: https://promotepollinators.org/about/history/. Accessed: 9 Dec 2022.

[CR130] Rader R, Bartomeus I, Garibaldi LA et al (2016) Non-bee insects are important contributors to global crop pollination. Proc Natl Acad Sci U S A 113:146–151. 10.1073/pnas.1517092112 26621730 10.1073/pnas.1517092112 PMC4711867

[CR131] Ratto F, Simmons BI, Spake R, et al (2018) Global importance of vertebrate pollinators for plant reproductive success: a meta‐analysis. 10.17863/CAM.21441

[CR132] Rech, A. R., K. Agostini, P. E. Oliveira & I. C. Machado (orgs.). (2014). Biologia da Polinização. Editora Projeto Cultural, Rio de Janeiro, 532p.

[CR133] Renzi A et al (2019) Evolução do controle biológico de insetos e pragas no setor canavieiro: uma análise na perspectiva econômica. Rev Agroecol Meio Ambient 12(2):459–485

[CR134] Requier F, Antúnez K, Morales CL, Sánchez PA, Castilhos D, Garrido PM, Giacobino A, Reynaldi FJ, Londoño JMR, Santos E, Garibaldi LA (2018) Trends in beekeeping and honey bee colony losses in Latin America. J Apic Res 57(5):657–662

[CR135] Requier, Fabrice ; Leyton, Malena Sibaja ; Morales, Carolina l. ; Garibaldi, Lucas A. ; Giacobino, Agostina ; Porrini, Martin Pablo ; Rosso-Londoño, Juan Manuel ; Velarde, Rodrigo A. ; Aignasse, Andrea ; Aldea-Sánchez, Patricia ; Allasino, Mariana Laura ; Arredondo, Daniela ; Audisio, Carina ; Cagnolo, Natalia Bulacio ; Basualdo, Marina ; Branchiccela, Belén ; Calderón, Rafael A. ; Castelli, Loreley ; Castilhos, Dayson ; Pires, C. S. S. ; et.al . First large-scale study reveals important losses of managed honey bee and stingless bee colonies in Latin America. Scientific Reports, v. 14, p. 1-15, 2024. 10.1038/s41598-024-59513-6 PMC11066017 38698037

[CR136] Ribas AP, Lourenço BHM, Araújo R, Vidigal MS, Alves BDC, Martins GF (2024) Exploring honey bee toxicological data as a proxy for assessing dimethoate sensitivity in stingless bees. Chemosphere 354:141652. 10.1016/j.chemosphere.2024.141652 38462182 10.1016/j.chemosphere.2024.141652

[CR137] Rocha Franco C, Pelaez V (2016) A (Des)Construção da Agenda Política de Controle dos Agrotóxicos no Brasil. Ambiente & Sociedade, São Paulo 19(3):215–232

[CR138] Rose, T., C. Kremen, A. Thrupp, B. Gemmill-Herren, B. Graub & N. AZZU. (2015). Policy Analysis Paper: Mainstreaming of Biodiversity and Ecosystem Services with a Focus on Pollination. FAO, Rome, x+53p.

[CR139] Sá, L. A. N., M. C. P. Y. Pessoa, G. J. de Moraes, J. S. Marinho Prado, S. S. Prado & R. M. de Vasconselos. (2016). Quarantine facilities and legal issues of the use of biocontrol agents in Brazil. Pesq. Agropec. Bras., Brasília, 51(5): 502-509.

[CR140] Santos GR (2012) Características, sistema de registros de produtos e concorrência no mercado de agrotóxicos no Brasil. Radar: Tecnologia, Produção e Comércio Exterior 20: [paginação não informada]. Instituto de Pesquisa Econômica Aplicada (Ipea). Disponível em: http://repositorio.ipea.gov.br/handle/11058/5371. Acesso em: 07 fev. 2026

[CR141] Schelske, O., L. Xing, C. Wong & F. Trepp. (2018). Making a beeline for disaster? The decline of pollinators puts agriculture at risk. Swiss Re Corporate Real Estate & Services, Zurich, 12p.

[CR142] Senado Federal (2013) Câmara dos deputados - Comissão debate mortandade de abelhas por causa do uso de agrotóxicos. Agência Câmara Notícias. https://www.camara.leg.br/noticias/408616-comissao-debate-mortandade-de-abelhas-por-causa-do-uso-de-agrotoxicos/. Accessed 10 Dec 2025

[CR143] Senado Federal. (2018). Audiência pública da Comissão de Agricultura e Reforma Agrária realizada em 20 de março de 2018 destinada a debater a importância dos insetos polinizadores para a agropecuária e os estudos recentes sobre o tema. Available at: https://legis.senado.leg.br/comissoes/reuniao?0&reuniao=7265&codcol=1307.

[CR144] Silva JM, Novato-Silva E, Faria HP, Pinheiro TMM (2005) Agrotóxico e trabalho: uma combinação perigosa para a saúde do trabalhador rural. Cienc Saude Coletiva 10(4):891–903

[CR145] Soares HM, Jacob CRO, Carvalho SM, Malaspina O (2015) Toxicity of Imidacloprid to the stingless bee *Scaptotrigona postica* Latreille, 1807 (Hymenoptera: Apidae). Bull Environ Contam Toxicol. 10.1007/s00128-015-1488-6 25666568 10.1007/s00128-015-1488-6

[CR146] Tang J, Wice J, Thomas VG, Kevan PG (2007) Assessment of Canadian federal and provincial legislation to conserve native and managed pollinators. Int J Biodivers Sci Manag 3(1):46–55

[CR147] Tavares DA, Roata TC, Carvalho SM, Silva-Zacarin ECM, Malaspina O (2015) In vitro effects of thiamethoxam on larvae of Africanized honey bee *Apis mellifera* (Hymenoptera: Apidae). Chemosphere 135:370–378 25985214 10.1016/j.chemosphere.2015.04.090

[CR148] Temperton VM, Buchmann N, Buisson E, et al (2019) Step back from the forest and step up to the Bonn Challenge: how a broad ecological perspective can promote successful landscape restoration. Restoration Ecology 27:705–719. 10.1111/rec.12989

[CR149] Tomé HVV, Ramos GS, Araújo MF, et al (2017) Agrochemical synergism imposes higher risk to Neotropical bees than to honeybees. R Soc Open Sci 4:160866. 10.1098/rsos.160866 10.1098/rsos.160866 PMC5319351 28280585

[CR150] Tosi, S., G. Burgio & J. C. Nieh. (2017a). A common neonicotinoid pesticide, thiamethoxam, impairs honey bee flight ability. Nature Scientific Reports 7(1201): 1-8. 10.1038/s41598-017-01361-8 PMC5430654 28446783

[CR151] Tosi, S., J. C. Nieh, F. Sgolastra, R. Cabbri & P. Medrycki. (2017b). Neonicotinoid pesticides and nutritional stress synergistically reduce survival in honey bees. Proc. R. Soc. B 284: 20171711. 10.1098/rspb.2017.1711 PMC5745400 29263280

[CR152] Valdovinos-Nuñes, G. R., J. J. G. Quesada-Euán, P. Ancona-Xiu, H. Moo-Valle & E. R. Sánchez. (2009). Comparative Toxicity of Pesticides to Stingless Bees (Hymenoptera: Apidae: Meliponini). J. Econ. Entomol. 102(5): 1737–1742. 10.1603/029.102.0502 19886436

[CR153] Vasconcelos, Y. (2018). Agrotóxicos em berlinda. Rev. Pesquisa Fapesp 271: 18-27.

[CR154] Viana BF, Boscolo D, Mariano Neto E et al (2012). How well do we understand landscape effects on pollinators and pollination services? J Pollen Ecol 7. 10.26786/1920-7603(2012)2

[CR155] Vidal MC, Amaral DFS, Nogueira JD, Mazzaro MAT (2021) Bioinsumos: a construção de um programa nacional para sustentabilidade do agro brasileiro. EARL 12(3):557–574

[CR156] Vieira, R. (2019). Liberou geral: Governo acelera a liberação de agrotóxicos. Revista Época (Editora Globo), Rio de Janeiro, 1100: 18-27.

[CR157] Witter, S., P. Nunes-Silva, B. Blochtein, B. B. Lisboa & V. L. Imperatriz-Fonseca. (2014). As abelhas e a agricultura [recurso eletrônico]. Editora Universitária da PUC-RS - EDIPUCRS, Porto Alegre, 143p.

[CR158] Wolowski, M., K. Agostini, A. R. Rech, I. G. Varassin, M. Maués, L. Freitas, L. T. Carneiro, R. O. Bueno, H. Consolaro, L. Carvalheiro, A. M. Saraiva, C. I. Silva & M. C. G. Padgurschi (orgs.). (2019). Relatório temático sobre polinização, polinizadores e produção de alimentos no Brasil. Editora Cubo, São Carlos.

[CR159] Wood SA, Karp DS, DeClerck F, Komatsu S, Jones NMK, Kremen C, Holland AD (2015) Functional traits in agriculture: agrobiodiversity and ecosystem services. Trends Ecol Evol 30:531–539. 10.1016/j.tree.2015.06.013 26190137 10.1016/j.tree.2015.06.013

[CR160] Wood TG, Goulson D (2017) The environmental risks of neonicotinoid pesticides: a review of the evidence post 2013. Environ Sci Pollut Res 24(21):17285–17325 10.1007/s11356-017-9240-x PMC5533829 28593544

[CR161] Woodcock BA, Garratt MPD, Powney GD et al (2019) Meta-analysis reveals that pollinator functional diversity and abundance enhance crop pollination and yield. Nat Commun 10:1481. 10.1038/s41467-019-09393-6 30931943 10.1038/s41467-019-09393-6 PMC6443707

[CR162] Yamamoto, Y., P. E. Oliveira & M. C. Gaglianone (coords.). (2014). Uso sustentável e restauração da diversidade dos polinizadores autóctones na agricultura e nos ecossistemas relacionados: planos de manejo. Fundo Brasileirao para a Biodiversidade - Funbio, Rio de Janeiro, 404p.

[CR163] Zhang W (2018) Global pesticide use: profile, trend, cost/benefit and more. Proc Int Acad Ecol Environ Sci 8(1):1–27

[CR164] Zurbuchen A, Landert L, Klaiber J, Müller A, Hein S, Dorn S (2010a) Maximum foraging ranges in solitary bees: only few individuals have the capability to cover long foraging distances. Biol Conserv 143:669–676

[CR165] Zurbuchen A, Cheesman S, Klaiber J, Muller A, Hein S, Dorn S (2010b) Long foraging distances impose high costs on offspring production in solitary bees. J Anim Ecol 79:674–681 20233258 10.1111/j.1365-2656.2010.01675.x

